# Supplementary material for: Utility of cell-free DNA concentrations and illness severity scores to predict survival in critically ill neonatal foals
Source: PLoS One. 2021 Apr 26;16(4):e0242635. doi: 10.1371/journal.pone.0242635 (PMC8075268; doi:10.1371/journal.pone.0242635)
Supplement: Attachment — Submitted filename: Response to Reviewers Round 2 4921.docx [file pone.0242635.s004.docx]

**Response to Reviewers: PONE-D-20-34767R1**

**The authors wish to once again thank the reviewers for the time and expertise that went into their critique. We believe the most recent edits to the manuscript have further improved the quality of the report and addressed your concerns.**

**Sincerely,**

**The authors**

Reviewer #1: Title: Utility of cell-free DNA concentrations and illness severity scores to predict survival in critically ill neonatal foals
#: PONE-D-20-34767R1
By: Colmer et al

The authors addressed major concerns raised in the initial submission of this manuscript. Other than minor grammatical errors or typos this reviewer is pleased with the response.

Minor:
While some findings remain intriguing, in particular the lack of statistical differences among groups, assuming that cell lysis from SIRS occurred in these foals, results could be real, supporting that cfDNA has limited use as an indicator of disease severity in critically ill equine neonates. Unfortunately, and as discussed, including animals with well documented tissue necrosis (e.g. colitis, pleuropneumonia, etc) as potential positive controls would have strengthen this manuscript (limitations).

**The authors agree that including animals with well-documented tissue necrosis (colitis or pleuropneumonia, etc) as positive controls could have strengthened the theory behind age influencing the lack of utility of cfDNA in the equine neonate discussed in this manuscript (line 341).**

It is unclear in the tables whether the reported SE is of the mean or the median (not the same). Clarification is important as SE of the median is often invalid because it makes assumption of normality (unless that information was from a normally distributed data set).
**We have clarified the data distribution and edited the table to show median and range.**

Reviewer #3: All the comments have been addressed and the manuscript is a welcome addition to the literature in this area. I have five very minor grammatical and typo comments below but these can almost be discarded. I congratulate the authors on a very nice manuscript.

Line 45- Slightly difficult to read. Maybe a comma around however may help?
Line 94- maybe saying 10 days old may make this clearer?
Line 171- Maybe reword to ‘by allowing them to come to…’
Line 181- change ares to are
Line 241- bacteremic is spelt incorrectly

**The authors appreciate and acknowledge the aforementioned suggestions in grammar and spelling, and the suggested alterations have been made.**
